# Supplementary material for: Synergistic Effect of Ribitol and Shikonin Promotes Apoptosis in Breast Cancer Cells
Source: Int J Mol Sci. 2025 Mar 15;26(6):2661. doi: 10.3390/ijms26062661 (PMC11942206; doi:10.3390/ijms26062661)
Supplement: Supplementary file 1 [file ijms-26-02661-s001.zip › ijms-3403596-supplementary.pdf]

Supplementary information:

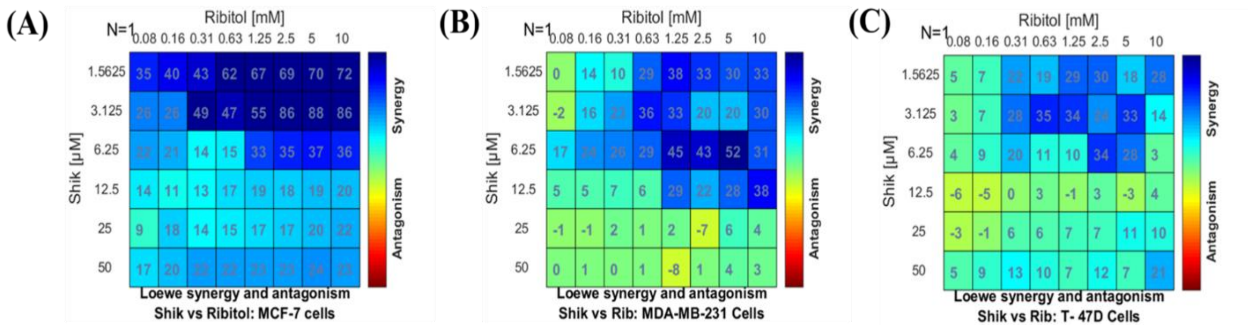

Supplementary Figure S1: Analysis of synergy between ribitol and shikonin by synergy matrix plots in breast cancer cells. (**A)** MCF-7 Cells, at the doses ranges from 1.56 µm-6.25 µm shikonin with 0.6mM-10mM ribitol (Blue color indicates synergy). The same analysis was also conducted for **(B)** MDA-MB-231 and **(C)** T-47D cells, where the results confirmed no synergistic effect of combined drugs at most of the doses (Green color) or a low degree of synergism. Regarding numerical value in synergy matrix, the synergy score, as the average excess response to drug interactions in cells, >20 indicates synergistic effect. For MCF-7 cells, the score was more than 40 (doses ranges from 1.56 µm-6.25 µm shikonin with 0.6mM-10mM ribitol) which indicates strong synergy between drugs. If the value was in negative, means there was no synergy between drugs.
